# Stream Noise, Hybridization, and Uncoupled Evolution of Call Traits in Two Lineages of Poison Frogs: *Oophaga histrionica* and *Oophaga lehmanni*

**DOI:** 10.1371/journal.pone.0077545

**Published:** 2013-10-23

**Authors:** Fernando Vargas-Salinas, Adolfo Amézquita

**Affiliations:** Department of Biological Sciences, Universidad de los Andes, Bogotá DC, Colombia; University of Saint-Etienne, France

## Abstract

According to the acoustic adaptation hypothesis, communication signals are evolutionary shaped in a way that minimizes its degradation and maximizes its contrast against the background noise. To compare the importance for call divergence of acoustic adaptation and hybridization, an evolutionary force allegedly promoting phenotypic variation, we compared the mate recognition signal of two species of poison frogs (*Oophaga histrionica* and *O. lehmanni*) at five localities: two (one per species) alongside noisy streams, two away from streams, and one interspecific hybrid. We recorded the calls of 47 males and characterized the microgeographic variation in their spectral and temporal features, measuring ambient noise level, body size, and body temperature as covariates. As predicted, frogs living in noisy habitats uttered high frequency calls and, in one species, were much smaller in size. These results support a previously unconsidered role of noise on streams as a selective force promoting an increase in call frequency and pleiotropic effects in body size. Regarding hybrid frogs, their calls overlapped in the signal space with the calls of one of the parental lineages. Our data support acoustic adaptation following two evolutionary routes but do not support the presumed role of hybridization in promoting phenotypic diversity.

## Introduction

Species exhibiting intraspecific geographic or microgeographic variation in habitat use offer excellent opportunities to understand the very initial steps of evolutionary divergence in auditory signals [1], [2]. After all, microgeographic variation is probably the minimum amount of evolution that can be detected in nature. Sensory drive is widely recognized as the selective pressure of habitat characteristics on the evolution of communication systems [3], [4]. Since habitats do vary geographically, local adaptations in mate recognition signals may promote reproductive isolation among populations and thereby speciation [5]. For instance, habitat color background is thought to promote the evolution of contrasting colors in dewlaps of visually displaying lizards *Anolis* [6] and in body parts of birds and cichlid fish [7], [8]. A form of sensory drive known as acoustic adaptation hypothesis (also termed signal structure hypothesis) implies that habitat characteristics such as background noise may evolutionarily shape auditory signals in a way that maximizes their contrast against the background noise [9]–[11]. The empirical evidence supporting adaptation in acoustic mating signals is surprisingly sparse compared to visual signals [6], [12]–[16].

Cross-breeding between taxa (hybridization) also can favor the evolution of communication signal diversity because hybrids often exhibit distinctive phenotypes [17], [18]. Distinctive communication traits in the “hybrid” offspring may help to maintain the integrity of hybrid lineages by reducing the chances of back crossbreeding with any parental species [18], [19] which may result in hybrid speciation [20]. Indeed, recent evidence suggests that hybridization can play an unsuspected important role in animal diversification [21]–[24]. However, hybridization events can also be costly if hybrid offspring is less adapted than parental individuals, thus promoting the evolution of strong premating isolation mechanisms (i.e. reinforcement [17], [25], [26]. Last but not least, hybridization has been also considered as a homogenizing force reversing initial divergence between lineages [21], [27]. Summing up, hybridization has been recognized as a widespread phenomenon in nature for decades [23], [28], but the variety of hybridization effects preclude strong generalizations about its role in the evolution of communication signals diversity.

The role of natural selection on the divergence of auditory communication signals has been compared to the role of other evolutionary forces such as sexual selection and genetic drift [29]–[32]. However, we are not aware of any attempt to simultaneously contrast the role of natural selection and hybridization in the evolution of an auditory signal. Whereas natural selection is expected to promote gradual divergence in communication signals, hybridization is considered an evolutionary mechanism capable of generating large phenotypic variation in short time intervals compared to natural selection [33], [34]. Thus, where both processes have recently occurred, one should expect either (1) a signal phenotype representing a tradeoff between adaptation and hybridization or (2) uncoupled evolution of signal characteristics, i.e. some signal traits will reflect the effect of natural selection whereas others will just represent the genetic consequences of hybridization.

Among vertebrates, poison frogs (Dendrobatidae) represent an increasingly important model in which to study the evolutionary biology of complex signals and its implications in speciation. They use multimodal (e.g. visual and acoustic) and multicomponent (e.g. color and pattern) communication signals [32], [35], [36] that are heritable ([37], [38], Amézquita, unpublished data) and often exhibit intraspecific geographic variation [32], [39]. Among the mechanisms allegedly promoting geographic variation in their complex signals are divergent female choice preferences, adaptation to local predators, mimetic processes, and genetic drift [32], [39]–[41]. We expect here to add to this knowledge by testing the simultaneous effect of natural selection represented by stream noise and hybridization in the evolution of an auditory signal.

The frogs *Oophaga histrionica* and *O. lehmanni* inhabit tropical wet forests where males usually establish territories, call, and mate near forest gaps and forest edges [42], [43]. At some localities, however, territorial males are heard from riparian forests, where the acoustic environment is strongly dominated by the noise produced by fast flowing streams. Streams produce a continuous broadband low-frequency noise, which should favor the evolution of high frequency calls in frogs as an adaptation that reduces masking interference by abiotic noise [44]–[46]. Hence, individuals of *O. histrionica* and *O. lehmanni* living alongside noisy streams should exhibit higher call frequencies than individuals living away from streams, where the broadband low-frequency noise is significantly lower in intensity. On the other hand, cross-breeding experiments and microsatellite analyses have shown that at least one population currently assigned to *O. histrionica* resulted from hybridization between *O. histrionica* and *O. lehmanni* [47]. Therefore, the hybrid population of *O. histrionica* could exhibit either intermediate or novel call traits relative to the parental lineages, perhaps distinctive enough to increase the probability of incipient speciation. Our aim here was to estimate the effect of stream noise and hybridization on microgeographic divergence of an acoustic mating signal. To reach this goal, we 1) characterized geographic variation on spectral and temporal traits of the advertisement calls of *O. histrionica* and *O. lehmanni*, and then used multivariate analyses to 2) compare each species' calls between individuals occurring at streams and away from them, and 3) compare the calls of a hybrid population against the call of the parental lineages.

## Materials and Methods

### Ethics statement

Procedures for recordings, capture and handling of live animals in the field were approved by the Colombian Ministry of Environment under the research permit 004 of July 27–2007. The Unidad Administrativa Especial de Parques Nacionales Naturales de Colombia and the Corporación Autónoma Regional del Valle del Cauca CVC gave us permission and logistic support for working in all study localities.

### Study species

**Figure 1:**
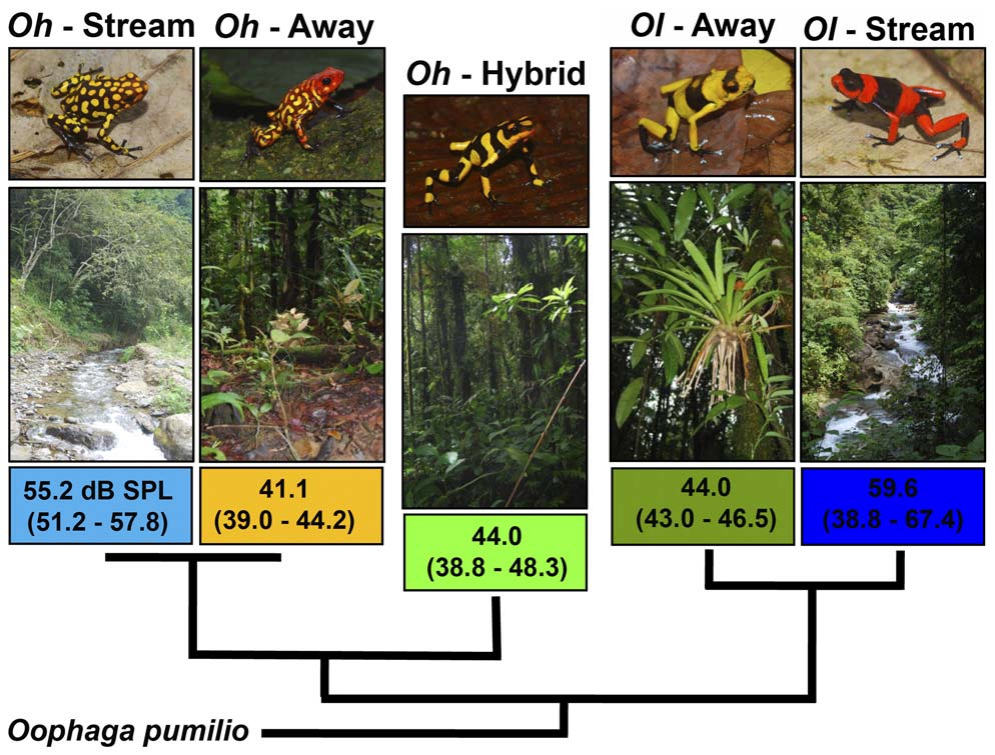
Study sampling scheme. Calls were recorded from two populations of *Oophaga histrionica*, two of *O. lehmanni* and a hybrid population between both species [47], which is currently assigned to *O. histrionica* (*Oh-*Hybrid). One population of each species (*Oh*-Stream, *Ol*-Stream) occurs along streams where environmental noise (median and range expressed in dB, re 20 µPa) is higher than for three other populations occurring away from streams (*Oh*-Away, *Oh-*Hybrid, *Ol*-Away). Tree topology from [47].

The poison frogs *Oophaga histrionica* and *O. lehmanni* (Dendrobatidae) inhabit tropical wet forests, on the pacific slope of the western Colombian Andes. Whereas *O. histrionica* is widely distributed between sea level and 1000 m elevation, *O. lehmanni* is scattered within less than 400 km^2^, between 550–1010 m elevation [43], [48]. Here we studied three populations currently assigned to *O. histrionica* and two populations to *O. lehmanni*, all of them in the Departamento del Valle del Cauca. We publish here the approximate position rather than the precise coordinates of the study sites because both species are heavily trafficked for the international pet market and *O. lehmanni* (Critically Endangered according to [49]) is almost extinct throughout its formerly known distribution range [50]. One population of each species occurs in riparian habitats (hereafter *Oh-*Stream, *Ol-*Stream) where the acoustic environment is highly influenced by high-intensity and low-frequency noise produced by rocky streams. Another population of each species occurs away from fast flowing streams (hereafter *Oh-*Away, *Ol-*Away), in less noisy habitats (Fig. 1). The third population of *O. histrionica* (hereafter *Oh*-Hybrid) has arisen from natural cross-breeding between the latter two populations of *O. histrionica* and *O. lehmanni* [47] and has been found predominantly away from noisy streams (Fig. 1).

### Recording and analysis of advertisement calls

Most individuals (40/47) were recorded in the field during ten field trips between April 2009 and September 2012; seven were recorded under lab conditions due to significant security concerns in the study area, which has been historically occupied by guerrillas. Calling males were generally found during daytime hours in elevated positions on the forest floor upon fallen trunks, leaves, and abundant leaflitter; in *Oh*-Stream, males called from the ground and from crevices in rocky areas along the streams. Once a calling male was located, we recorded its advertisement calls by positioning a unidirectional microphone (Sennheiser K6/ME66), connected to a digital recorder (Marantz PMD660), 30–150 cm from a calling male. Immediately after recording we measured male body temperature to the nearest 0.1°C with an infrared thermometer (Oakton model 35629) and captured him to measure body size (snout-vent length, SVL) to the nearest 0.1 mm with a digital caliper. At the end of each recording session, we measured ambient noise level (maximum value during 10 s) at the calling position to the nearest dB (re 20 µPa) with a sound level meter Roline RO-1350.

Digital recordings were analyzed with Raven 1.4 sound analysis software [51]. The terminology and procedures for measuring call traits were based on [52]. From oscillographic representations of the call, we measured pulse cycle duration (pulse duration + interpulse interval duration), call duration, number of pulses per call and then calculated pulse repetition rate; from spectrograms we measured peak frequency as the call frequency with the highest energy content. Because call traits such as peak frequency and pulse cycle duration can vary within a single call in *O. histrionica* and *O. lehmanni*, we repeated these measurements in three pulses located at the initial, middle and final portions of a single call and then used the corresponding average values for further analyses. From power spectra (Window: Blackman, DFT: 2048 samples), we measured peak frequency. For each call parameter, the average of measurements taken on three calls per male was used as the smallest unit of statistical analysis.

### Statistical analyses

Since sound pressure levels were measured in dB, which represent a logarithmic scale, the calculation of the average values and further statistical analyses were conducted after converting dB values to a linear scale (pressure, Pa). Differences in ambient noise level among populations were tested with an Analysis of Variance ANOVA using significant level of α<0.05. Since the other measured acoustic traits are usually intercorrelated, we reduced redundancy by conducting a principal component analysis (PCA) with Varimax-rotation. Because body temperature may affect calling performance and thereby acoustic trait values, we removed its effect by conducting linear regression on PCAs, and then used the regression residuals as new temperature-independent acoustic traits in subsequent statistical analyses. To estimate the degree of acoustic divergence among the five studied populations we ran a canonical discriminant analysis. To test whether abiotic noise originated from streams was correlated with high call frequency in *Oh*-Stream and *Ol-*Stream, we ran an ANOVA. Because part of the call divergence could be merely explained by co-variation in body size, we repeated the ANOVA after removing concomitant variation in male body size [37], [53]. To test for intra-locality relationships between ambient noise level and call characteristics (PCAs after statistically controlling effect of temperature) we used an analysis of covariance (ANCOVA).

## Results

**Figure 2:**
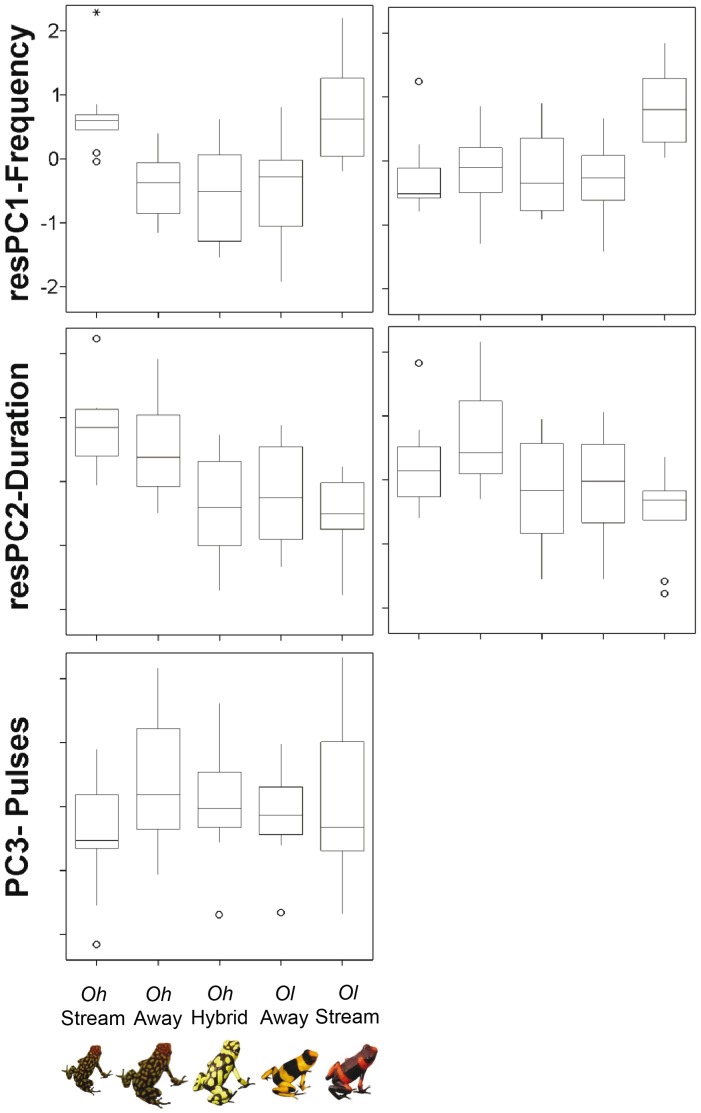
Geographic variation in advertisement calls among populations of *Oophaga histrionica* (*Oh*-Stream, *Oh*-Away), *O. lehmanni* (*Ol*-Away, *Ol*-Stream), and a hybrid population between both species. Boxplots represent principal component scores (see Table 1) that summarize co-variation in original call traits after controlling for temperature (left column), or temperature and body size (right column), except for PC3-Pulse number which was not correlated with either variable. Sample size: *Oh*-Stream  = 9 males, *Oh*-Away  = 8, *Oh-*Hybrid  = 8, *Ol*-Away  = 12, and *Ol*-Stream  = 10.

As expected, we detected significant non-overlapping differences in environmental noise between the habitats of streamside frogs (*Oh-*Stream and *Ol-*Stream) and frogs living away from streams (*Oh-*Away, *Ol-*Away, and *Oh-*Hybrid) (F = 20.546, DF = 4, P<0.001; Fig. 1). Variation in the measured call traits (Table S1) was successfully summarized by three principal components (Table 1) mainly correlated with call frequency (PC1), call duration (PC2), and pulse number (PC3). After removing the effect of temperature, we detected significant microgeographic divergence in spectral (ANOVA, resPC1-Frequency: F = 7.995, P<0.001) and one of the temporal call features (resPC2-Duration: F = 5.786, P = 0.001; PC3: F = 0.799, P = 0.533) (Fig. 2). Raw data of call features for every frog are available on request to authors.

**Table 1:** Principal component analysis summarizing variation in advertisement calls of *Oophaga histrionica* (*Oh*-Stream, *Oh*-Away, *Oh-*Hybrid) and *O. lehmanni* (*Ol*-Away, *Ol*-Stream).

	Principal component		
Call variables	PC1	PC2	PC3
Median peak frequency (Hz)	0.954	−0.188	0.072
Final peak frequency (Hz)	0.922	−0.062	0.036
Initial peak frequency (Hz)	0.888	−0.182	0.175
Call duration (ms)	−0.107	0.941	−0.267
Median cycle duration (ms)	−0.043	0.784	0.125
Final cycle duration (ms)	−0.353	0.778	−0.079
Pulse number	−0.029	0.348	−0.883
Initial cycle duration (ms)	0.178	0.170	0.876
Eigenvalue	3.556	1.717	1.452
% of variance explained	34.010	29.094	20.962

The highest loadings for each principal component are Value >0.7.

**Figure 3:**
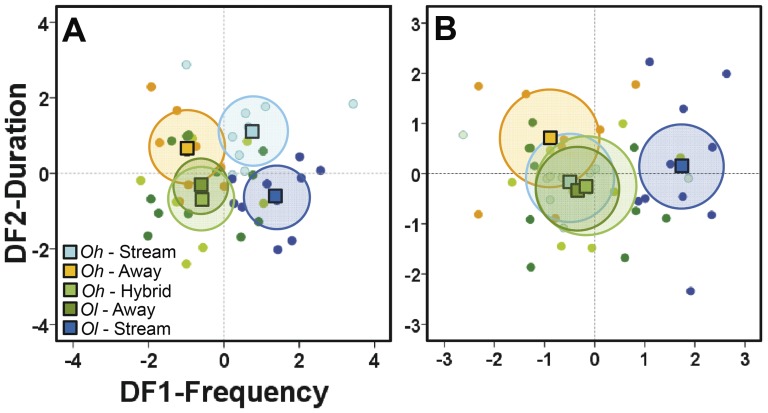
Variation in advertisement call among populations. Discriminant plots of microgeographic differences in advertisement calls of two populations of *Oophaga histrionica* (*Oh*-Stream, *Oh*-Away), two of *O. lehmanni* (*Ol*-Away, *Ol*-Stream), and a hybrid population between both species (*Oh*-Hybrid). The first discriminant function mainly represents call frequency whereas the second represents call duration (see also Table 2 for values). A: After controlling for covariation in body temperature. B. After controlling for covariation in body temperature and body size. Dots represent recorded males, squares represent the bivariate centroids for each population, and the shadowed ellipses encompass the 95% confidence limit for each centroid. Populations that are significantly different should have non-intersecting ellipses.

**Figure 4:**
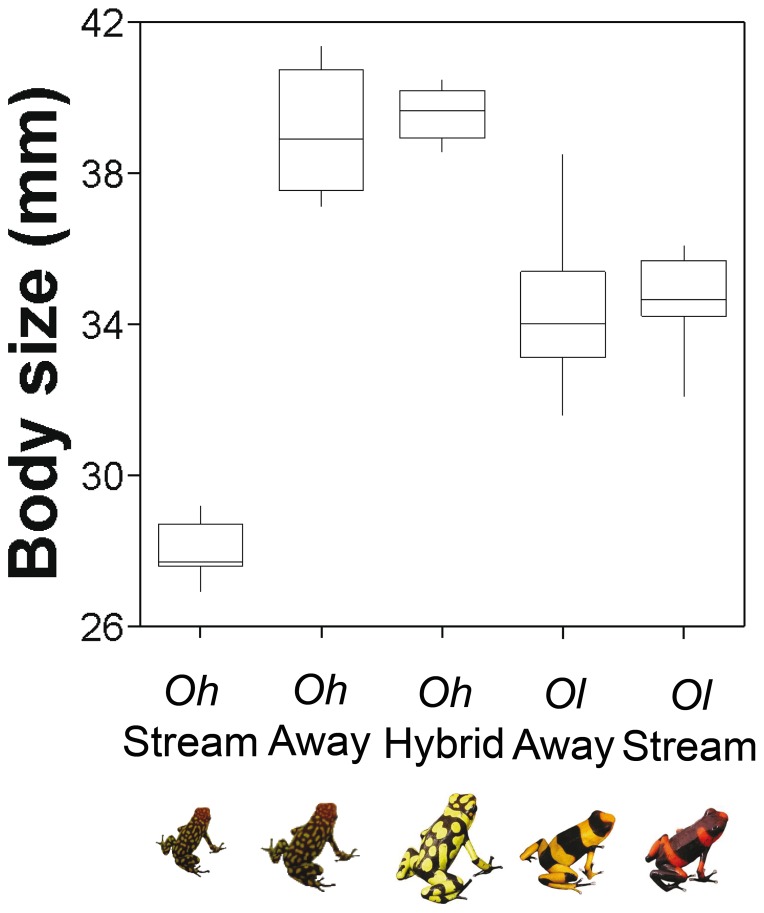
Geographic variation in body size. Difference in body size (Snout-vent length, SVL) among populations of *Oophaga histrionica* (*Oh*-Stream, *Oh*-Away), *O. lehmanni* (*Ol*-Away, *Ol*-Stream), and a hybrid population between both species (*Oh*-Hybrid). Sample size: *Oh*-Stream  = 9 males, *Oh*-Away  = 8, *Oh-*Hybrid  = 8, *Ol*-Away  = 12, *Ol*-Stream  = 10.

The first discriminant function (Table 2), mostly related to resPC1-Frequency, explained 58.8% of call variation. It separated very well two populations with high frequency calls (*Oh-*Stream and *Ol-*Stream) from three populations with low frequency calls (*Oh-*Away, *Ol-*Away and *Oh-*Hybrid) (Fig. 3A). The second discriminant function, mostly related to resPC2-Duration, explained an additional 37.1% of the variation. Along the corresponding axis, it tended to separate two populations with relatively long calls (*Oh-*Stream and *Oh-*Away) from three populations with shorter calls (*Ol-*Stream, *Ol-*Away, and *Oh-*Hybrid). Considering both functions, the calls of hybrid frogs, *Oh-*Hybrid, overlapped almost completely with the calls of *Ol-*Away. Since populations differed in average body size (F = 91.52, DF = 4, P<0.001; Fig. 4), we repeated the discriminant analysis after controlling for both temperature and body size effects (Multiple regression, PC1: R^2^ = 0.431, F = 16,673, P<0.001; PC2: R^2^ = 0.278, F = 8.453, P = 0.001). As a result (Table 2), one of the riparian populations (*Oh-*Stream) moved towards the signal spaces of *Ol-*Away and *Oh-*Hybrid and overlapped completely within them (Fig. 3B). The other riparian population (*Ol-*Stream) remained statistically distinguishable from any other population, because of its high frequency calls. There was no intrapopulation relationship between ambient noise level and any of the call characteristics (resPC1-Frequency: Ancova F = 0.572, DF = 1, P = 0.455, N = 40; resPC2-Duration: F = 0.682, DF = 1, P = 0.415; PC3-Pulse number: F = 0.214, DF = 1, P = 0.647).

**Table 2:** Discriminant analyses to predict population membership of male advertisement calls of *Oophaga histrionica* and *O. lehmanni*.

	Discriminant function		
Call variables	1	2	3
Eigenvalue	0.873 (0.960)	0.551 (0.141)	0.060 (0.044)
% of variance explained	58.8 (83.8)	37.1 (12.3)	4.0 (3.9)
Statistic significance	<0.001 (<0.001)	0.002 (0.288)	0.294 (0.402)
resPC1 – Frequency	0.894 (0.732)	0.324 (0.632)	0.309 (−0.257)
resPC2 – Duration	−0.117 (−0.457)	0.989 (0.722)	0.090 (−0.519)
PC3 – Pulse number	−0.080 (0.057)	−0.149 (0.544)	0.986 (0.837)

Values before and after (in parentheses) controlling for concomitant variation in body size (see also Fig. 3). In the lower half, the highest discrimination coefficients represent the variables with the highest influence on each discriminant function.

## Discussion

Our results demonstrate uncoupled divergence in call traits among the studied populations. Concerning the spectral call traits, populations were grouped independently of their phylogenetic relationship, but according to similarities on their habitat acoustic environment. In contrast, concerning temporal call traits, populations were grouped according to their phylogenetic affinity rather than their acoustic environment. Hybridization did not appear to promote call diversity in our study system.

**Figure 5:**
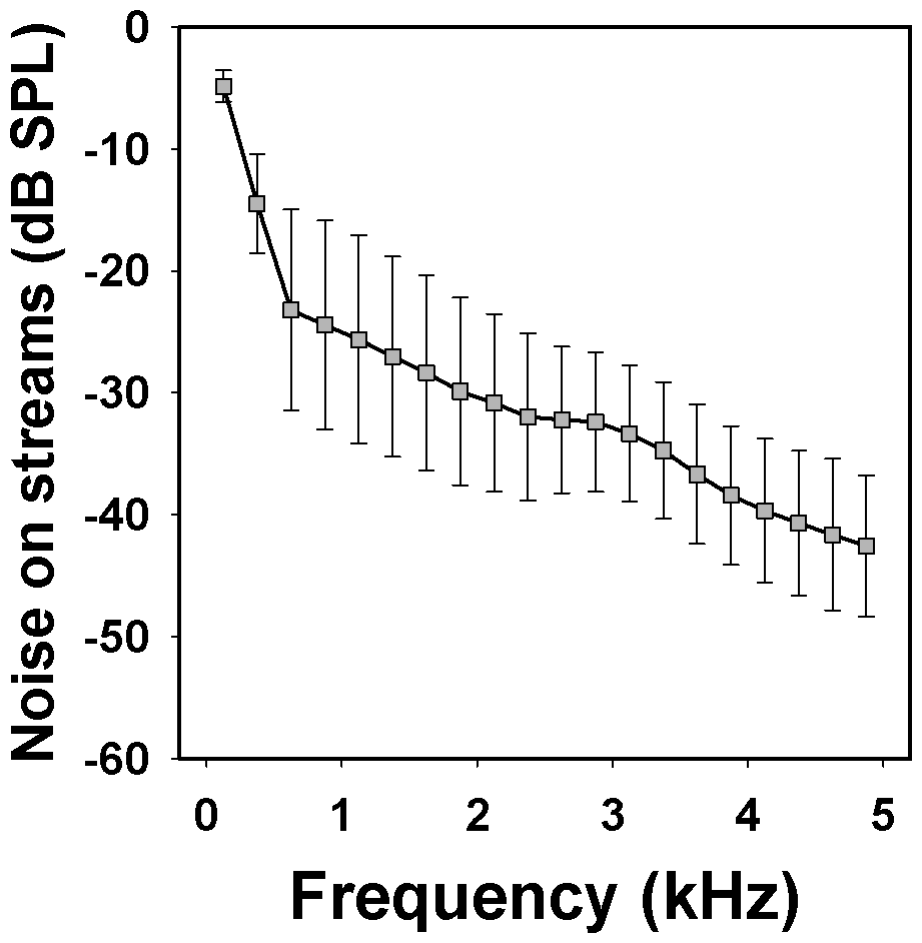
Power spectra of the abiotic noise on streams. Symbols denote mean values (squares) and standard deviations based on eight recordings (Window: Blackman, DFT: 2048 samples).

Supporting our first prediction and the acoustic adaptation hypothesis, males from the two populations occurring along noisy streams (one per species, *Oh-*Stream and *Ol-*Stream) produced calls with higher frequency than males living away from streams. The pattern suggests parallel adaptation in both species. Since stream noise contains most of its energy at low frequencies ([44], [45], [54]; see Fig. 5), calling at higher frequencies should increase the signal-to-noise ratio and thereby the probability of being detected and recognized by conespecific receivers [55]. Vegetation complexity is also thought to affect the evolution of auditory signals, e.g. in birds [26], [56]. However, the empirical evidence supporting this effect in anurans is still weak and most comparisons have been made between populations inhabiting strikingly different habitats, such as open and forested areas [57]–[59]. Although we did not measure habitat complexity, variability between frogs in the microhabitat around calling perches appeared to be much higher than the corresponding differences between populations. In contrast, noise is obviously a more continuous and predictable environmental pressure, especially where frogs are restricted to narrow forest bands along the streams.

An upward shift in call frequency may have been evolutionary attained in two ways. First, call frequency is largely determined by vocal cord mass, which, in turn, is often correlated with male body size [53], [60]. Thus, stream noise in riparian habitats may actually confer a selective advantage to small males. Males of *Oh-*Stream are smaller than any other studied male (Fig. 4) and, once we statistically removed the negative effect of body size on call frequency, their calls are not longer distinguishable from the calls of males living away from streams (Fig. 3). Of course, the pleiotropical link between call frequency and body size can confound the effect of selection on either trait [37]. Numerous selective pressures are thought to act mainly on body size and pleiotropically affect call frequency [61]–[63]. Conversely, selective pressures on detection and recognition of mate recognition signals could be strong enough to promote concomitant changes in body size, given their tremendous importance for fitness [64], [65]. Interestingly, the latter possibility has been very rarely explored [31], [37].

The upward shift in call frequency may also have arisen from morphological variation in the vocal apparatus that is not pleiotropically linked to body size. A shift in call frequency without concomitant variation in body size was observed in *O. lehmanni* (*Ol-*Stream). Their calls remained distinguishable in frequency from the calls of any other population even after statistically removing the negative effect of body size (Fig. 3B). A morphological analysis of the frogs' vocal apparatus is beyond the scope of this study; however, previous studies indicate that novel fibrous masses adhered to the vocal cords may effectively increase their mass and thereby lead to the evolution of low frequency calls [64]. Another possibility, much less explored, is the modification of vocal chords' tension without concomitant effects on their mass.

Increasing call frequency implies that the acoustic signal is more locatable by receivers, but it propagates at a shorter distances [37]. It is assumed that signals covering larger distances can increase mating chances to the emissary because it can be detected by a higher number of females. However, it is possible that calls of many frog species are adapted for ease of localization at short distances instead that for long range propagation [65]. In fact, several streamside breeding anurans are characterized by weak calls and/or lack of auditory signals [66], [67]. Increasing signal's call frequency also can imply higher scattering and excess attenuation compared with lower call frequency signals [37]. However, recent evidence indicates that vegetation density affects the temporal rather than the spectral features of anuran calls [68], and studies on anuran assemblages have generally failed to find evidence supporting a role of habitat structure in the evolution of call traits [57], [59], [61], [69].

Our data failed to support the second prediction: hybrid calls were not intermediate between the parental lineages' calls nor were they distinctive enough to increase the probability of incipient speciation. Although both species' calls could be differentiated on the basis of their temporal parameters (call duration, pulses per call), *Oh-*Hybrid calls overlapped almost completely with *Ol-*Away calls (Fig. 3). Studies in other taxa have also found that hybrid calls are very similar to those produced by one of the parental species [65], [70]. The absence of distinctive call traits in the hybrid population implies that the hybrid population may well continue back-crossing with one of its parental species [47]. Alternatively, mate recognition might depend upon a combination of auditory signals and visual cues (color pattern) as has been suggested for *Oophaga pumilio*, a phylogenetically close taxon [40], [71]. This possibility, however, remains to be tested.

Summing up, our data support the hypothesis that stream noise has favored the evolution of high frequency calls in the poison frogs *O. histrionica* and *O. lehmanni* on a microgeographic scale. The evolutionary route, however, apparently differed between the species: it implied pleiotropical differences in body size in *O. histrionica* but not in *O. lehmanni*. Stream noise appears to be a selective force strong enough to promote microgeographic divergence in calls despite the counteracting effect of genetic flow at the microgeographic scale [47]. The potential for speciation of this environmentally driven signal diversification deserves further study.

## Supporting Information

Table S1 Statistical summary of advertisement call traits, made for the studied populations of poison frogs: *Oophaga histrionica* (*Oh*-Stream, *Oh*-Away), *O. lehmanni* (*Ol*-Away, *Ol*-Stream), and a hybrid population between both species (*Oh*-Hybrid). Values are Mean ± standard deviation.
(DOC)

## References

[1] Wilczynski W, Ryan MJ (1999) Geographic variation in animal communication systems. In: Foster SA, Endler JA, editors. Geographic variation in behavior. New York, USA: Oxford University Press. pp. 234–261.

[2] Rundle DH, Boughman JW (2010) Behavioral ecology and speciation. In: Westneat DF, Fox CW, editors. Evolutionary behavioural ecology.New York, USA: Oxford University Press Inc. pp. 471–487.

[3] Endler JA (1992) Signals, signal conditions and the direction of evolution. Am Nat 139: S125–S153.

[4] Endler JA (1993) Some general comments on the evolution and design of animal communication systems. Phil Trans R Soc B 340: 215–225. 8101656 10.1098/rstb.1993.0060

[5] Boughman JW (2002) How sensory drive can promote speciation. Trends Ecol Evol 17: 571–577.

[6] Fleishman LJ (2000) Signal function, signal efficiency and the evolution of anoline lizard dewlap color. In: Espmark Y, Amundsen T, Rosenqvist G, editors. Animal signals: signalling and signal design in animal communication. Trondheim, Norway: Tapir Academic Press. pp. 209–236.

[7] Marchetti K (1993) Dark habitats and bright birds illustrate the role of the environment in species divergence. Nature 362: 149–152.

[8] Seehausen O, Terai Y, Magalhaes IS, Carleton KL, Mrosso HDJ, et al (2008) Speciation through sensory drive in cichlid fish. Nature 455: 620–U623. 18833272 10.1038/nature07285

[9] Chappuis C (1971) Un example de l′influence du milieu sur les emissions vocals des oiseaux: l′evolution des chants en foret equatoriale. Terre et Vie 118: 183–202.

[10] Morton ES (1975) Ecological sources of selection on avian sounds. Am Nat 109: 17–34.

[11] Ryan MJ, Brenowitz EA (1985) The role of body size, phylogeny, and ambient noise in the evolution of bird song. Am Nat 126: 87–100.

[12] Fleishman LJ (1992) The influence of the sensory system and the environment on motion patterns in the visual displays of anoline lizards and other vertebrates. Am Nat 139: S36–S61.

[13] Boughman JW (2001) Divergent sexual selection enhances reproductive isolation in sticklebacks. Nature 411: 944–948. 11418857 10.1038/35082064

[14] Gómez D, Thery M (2004) Influence of ambient light on the evolution of colour signals: comparative analysis of a Neotropical rainforest bird community. Ecol Lett 2004: 279–284.

[15] Ord TJ, Peters RA, Clucas B, Stamps JA (2007) Lizards speed up visual displays in noisy motion habitats. Proc. R. Soc. B. 274: 1057–1062. 10.1098/rspb.2006.0263 PMC2124473 17264059

[16] Nemeth E, Brumm H (2010) Birds and anthropogenic noise: are urban songs adaptive? Am Nat 176: 465–475. 20712517 10.1086/656275

[17] Coyne JA, Orr HA (1997) Patterns of speciation in *Drosophila* revisited. Evolution 51: 295–303. 28568795 10.1111/j.1558-5646.1997.tb02412.x

[18] Price T (2007) Speciation in birds. Greenwood Village Colorado USA: Roberts and Company. 480 p.

[19] Gerhardt HC, Guttman SI, Karlin AA (1980) Natural hybrids between *Hyla cinerea* and *Hyla gratiosa*: morphology, vocalization and electrophoretic analysis. Copeia 1980: 577–584.

[20] Mavárez J, Salazar CA, Bermingham E, Salcedo C, Jiggins CD, et al (2006) Speciation by hybridization in *Heliconius* butterflies. Nature 441: 868–871. 16778888 10.1038/nature04738

[21] Seehausen O (2004) Hybridization and adaptive radiation. Trends Ecol Evol 19: 198–207. 16701254 10.1016/j.tree.2004.01.003

[22] Mallet J (2007) Hybrid speciation. Nature 446: 279–283. 17361174 10.1038/nature05706

[23] Mallet J (2008) Hybridization, ecological races and the nature of species: empirical evidence for the ease of speciation. Phil Trans R Soc B 363: 2971–2986. 18579473 10.1098/rstb.2008.0081 PMC2607318

[24] Larsen PA, Marchan-Rivadeneira MR, Baker RJ (2010) Natural hybridization generates mammalian lineage with species characteristics. Proc Natl Acad Sci USA 107: 11447–11452. 20534512 10.1073/pnas.1000133107 PMC2895066

[25] Sartre GP, Moum T, Bures S, Kral M, Adamjan M, et al (1997) A sexually selected character displacement in flycatchers reinforces premating isolation. Nature 387: 589–591.

[26] Seddon N (2005) Ecological adaptation and species recognition drives vocal evolution in neotropical suboscine birds. Evolution 59: 200–215. 15792239

[27] Seehausen O, Takimoto G, Roy D, Jokela J (2007) Speciation reversal and biodiversity dynamics with changing hybridization environments. Mol Ecol 17: 30–44. 18034800 10.1111/j.1365-294X.2007.03529.x

[28] Anderson E (1949) Introgressive hybridization. New York: John Wiley and Sons. 109 p.

[29] Ryan MJ, Rand AS, Weigt LA (1996) Allozyme and advertisement call variation in the túngara frog, *Physalaemus pustulosus* . Evolution 50: 2435–2453. 28565650 10.1111/j.1558-5646.1996.tb03630.x

[30] Irwin DE (2000) Song variation in an avian ring species. Evolution 54: 998–1010. 10937272 10.1111/j.0014-3820.2000.tb00099.x

[31] Lougheed SC, Austin JD, Bogart JP, Boag PT, Chek A (2006) Multi-character perspectives on the evolution of intraspecific differentiation in a neotropical hylid frog. BMC Evol Biol 6: 1–16. 16539709 10.1186/1471-2148-6-23 PMC1434785

[32] Amézquita A, Lima AP, Jehle R, Castellanos L, Ramos O, et al (2009) Calls, colours, shape, and genes: a multi-trait approach to the study of geographic variation in the Amazonian frog *Allobates femoralis* . Biol J Linn Soc 98: 826–838.

[33] Coyne JA, Orr HA (2004) Speciation. Sunderland Massachusetts USA: Sinauer Associates Inc. 545 p.

[34] Wiens JA (2004) What is speciation and how should we study it? Am Nat 163: 914–923. 15266388 10.1086/386552

[35] Narins PM, Hödl W, Grabul DS (2003) Bimodal signal requisite for agonistic behavior in a dart-poison frog, *Epipedobates femoralis* . Proc Natl Acad Sci USA 100: 577–580. 12515862 10.1073/pnas.0237165100 PMC141038

[36] De Luna AG, Hödl W, Amézquita A (2010) Colour, size and movement as visual subcomponents in multimodal communication by the frog *Allobates femoralis* . Anim Behav 79: 739–745.

[37] Gerhardt HC, Huber F (2002) Acoustic communication in insects and anurans: common problems and diverse solutions. Chicago: The University of Chicago Press. 542 p.

[38] Summers K, Cronin TW, Kennedy T (2004) Cross-breeding of distinct color morphs of the strawberry poison frog (*Dendrobates pumilio*) from the Bocas del Toro Archipelago, Panamá. J Herpetol 38: 1–8.

[39] Wollenberg KC, Lötters S, Mora-Ferrer C, Veith M (2008) Disentangling composite colour patterns in a poison frog species. Biol J Linn Soc 93: 433–444.

[40] Summers K, Symula R, Clough M, Cronin T (1999) Visual mate choice in poison frogs. Proc. R. Soc. B. 266: 2141–2145. 10.1098/rspb.1999.0900 PMC1690338 10649631

[41] Roberts JL, Brown JL, Schulte R, Arizabal W, Summers K (2007) Rapid diversification of colouration among populations of a poison frog isolated on sky peninsulas in the central cordilleras of Perú. J Biogeogr 34: 417–426.

[42] Silverstone PA (1973) Observations on the behavior and ecology of a Colombian poison-arrow frog the kokoe-pa *Dendrobates histrionicus* . Herpetologica 29: 295–301.

[43] Silverstone PA (1975) A revision of the poison-arrow frogs of the genus *Dendrobates* Wagler. Nat Hist Bull Angeles County Sci Bull 21: 1–55.

[44] Dubois A, Martens J (1984) A case of possible vocal convergence between frogs and a bird in Himalayan torrents. J Ornithol 125: 455–463.

[45] Narins PM, Feng AS, Lin W, Schnitzler HU, Denzinger A, et al (2004) Old World frog and bird vocalizations contain prominent ultrasonic harmonics. J Acous Soc Am 115: 910–913. 10.1121/1.1636851 15000202

[46] Feng AS, Narins PM, Xu CH, Lin WY, Yu ZL, et al (2006) Ultrasonic communication in frogs. Nature 440: 333–336. 16541072 10.1038/nature04416

[47] Medina I, Wang I, Salazar CA, Amézquita A (2013) Hybridization promotes color polymorphism in the aposematic harlequin poison frog, *Oophaga histrionica*. Ecol Evol in press. 10.1002/ece3.794 PMC3856739 24340180

[48] Lötters S, Jungfer K-H, Henkel FW, Schmidt W (2007) Poison frogs: biology, species and captive husbandry. FrankfurtGermany: Chimaira. 1–668 p.

[49] Castro-Herrera F, Amézquita A (2004) Rana venenosa de Lehmann *Dendrobates lehmanni*. In: Rueda-Almonacid JV, Lynch JD, Amézquita A. editors. Libro rojo de anfibios de Colombia. Serie de libros rojos de especies amenazadas de Colombia. Bogotá, Colombia: Conservación Internacional Colombia, Instituto de Ciencias Naturales Universidad de Colombia, Ministerio del Medio Ambiente. pp. 162–167.

[50] Velázquez EBE, Corredor LG, Velazco J, Amézquita A (2009) Evaluación del estado de conservación de la rana venenosa de Lehmann (*Oophaga lehmanni*), con fines de establecer una reserva natural para su protección. Santiago de CaliColombia: Corporación Autónoma del Valle del Cauca CVC, Wildlife Conservation Society WCS, Fundación CREA Zoológico de Cali. 32 p.

[51] Bioacoustics Research Program (2011) Raven Pro: Interactive Sound Analysis Software (Version 1.4) [Computer software]. Ithaca, NY: The Cornell Lab of Ornithology. Available from http://www.birds.cornell.edu/raven.

[52] Cocroft RB, Ryan MJ (1995) Patterns of advertisement call evolution in toads and chorus frogs. Anim Behav 49: 283–303.

[53] Martin WF (1972) Evolution of vocalization in the genus *Bufo* In: Blair WF, editor. Evolution in the genus *Bufo* Austin: University of Texas Press.pp. 279–309.

[54] Hödl W, Amézquita A (2001) Visual signaling in anuran amphibians. In: Ryan MJ, editor. Anuran communication. Washington DC: Smithsonian Institution Press. pp. 121–141.

[55] Grafe TH, Preininger D, Sztatecsny M, Kasah R, Dehling JM, et al (2012) Multimodal communication in a noisy environment: a case study of the bornean rock frog *Staurois parvus* . Plos ONE 7: 1–8. 10.1371/journal.pone.0037965 PMC3360010 22655089

[56] Wiley RH, Richards DG (1982) Adaptations for acoustic communication in birds: sound transmission and signal detection. In: Kroodsma DE, Miller EH, Ouellet H, editors. Acoustic communication in birds. Volume 1. Ithaca New York: Cornell University Press. pp. 131–181.

[57] Bosch J, De la Riva I (2004) Are frog calls modulated by the environment? An analysis with anuran species from Bolivia. Can J Zool 82: 880–888.

[58] Ryan MJ, Wilczynski W (1991) Evolution of intraspecific variation in the advertisement call of a cricket frog (*Acris crepitans* Hylidae). Biol J Linn Soc 44: 249–272.

[59] Zimmermann BL (1983) A comparison of structural features of calls of open and forest habitat frog species in the central Amazon. Herpetologica 39: 235–246.

[60] McClelland BE, Wilczynski W, Ryan MJ (1996) Correlations between call characteristics and morphology in male cricket frogs (*Acris crepitans*). J Exp Biol 199: 1907–1919. 8831143 10.1242/jeb.199.9.1907

[61] Ryan MJ (1987) Constraints and patterns in the evolution of anuran acoustic communication. In: Fritzsch B, Ryan MJ, Wilczynsky W, Hetherington TE, Walkowiak W, editors. The evolution of amphibian auditory system. Austin Texas, USA: Wiley Interscience. pp. 637–677.

[62] Kime NM, Turner WR, Ryan MJ (2000) The transmission of advertisement calls in Central American frogs. Behav Ecol 11: 71–83.

[63] Meenderink SWF, Kits M, Narins PM (2010) Frequency matching of vocalizations to inner-ear sensitivity along an altitudinal gradient in the coqui frog. Biol Lett 6: 278–281. 19939848 10.1098/rsbl.2009.0763 PMC2865046

[64] Ryan MJ, Drewes RC (1990) Vocal morphology of the *Physalaemus pustulosus* species group (Family Leptodactylidae): morphological response to sexual selection for complex calls. Biol J Linn Soc 40: 37–52.

[65] Wells KD (2007) Ecology and behavior of amphibians. Chicago & London: The University of Chicago Press. 1400 p.

[66] Duellman WE, Trueb L (1986) Biology of amphibians. New York: McGraw Hill Book Co. 1–670 p.

[67] Duellman WE (2001) The Hylid frogs of Middle America. IthacaNew YorkUSA: Natural History Museum of the University of Kansas. 1159 p.

[68] Ziegler L, Arim M, Narins PM (2011) Linking amphibian call structure to the environment: the interplay between phenotypic flexibility and individual attributes. Behav Ecol 22: 520–526. 22479134 10.1093/beheco/arr011 PMC3078827

[69] Penna M, Solis R (1998) Frog call intensities and sound propagation in the South American temperate forest region. Behav Ecol Sociobiol 42: 371–381.

[70] Schneider H, Sinsch U (2007) Contributions of bioacoustics to the taxonomy of the anura. In: Heatwole H, Tyler M, editors. Amphibian biology. Volume 7. Australia: Surrey Beatty & sons. pp. 2893–2933.

[71] Maan ME, Cummings ME (2008) Female preferences for aposematic signal components in a polymorphic poison frog. Evolution 62: 2334–2345. 18616568 10.1111/j.1558-5646.2008.00454.x

