# Supplementary material for: Stream Noise, Hybridization, and Uncoupled Evolution of Call Traits in Two Lineages of Poison Frogs: Oophaga histrionica and Oophaga lehmanni
Source: PLoS One. 2013 Oct 23;8(10):e77545. doi: 10.1371/journal.pone.0077545 (PMC3806806; doi:10.1371/journal.pone.0077545)
Supplement: Table S1 — Statistical summary of advertisement call traits, made for the studied populations of poison frogs: Oophaga histrionica (Oh-Stream, Oh-Away), O. lehmanni (Ol-Away, Ol-Stream), and a hybrid population between both species (Oh-Hybrid). Values are Mean ± standard deviation. (DOC) [file pone.0077545.s001.doc]

**Table S1.** Statistical summary of advertisement call traits, made for the studied populations of poison frogs: *Oophaga histrionica* (*Oh*-Stream, *Oh*-Away, *Oh-*Hybrid), and *O. lehmanni* (*Ol*-Away, *Ol*-Stream). Values are Mean ± standard deviation.

| **Population** | **Call duration (ms)** | **Pulse number** | **Pulse repetition rate (pulses/s)** | **Initial cycle duration (ms)** | **Median cycle duration (ms)** | **Final cycle duration (ms)** | **Initial peak frequency (Hz)** | **Median peak frequency (Hz)** | **Final peak frequency (Hz)** |
| --- | --- | --- | --- | --- | --- | --- | --- | --- | --- |
| *Oh*-Stream | 178 ± 26.78 | 33.96 ± 6.48 | 0.195 ± 0.025 | 5.580 ± 2,025 | 5.358 ± 0.992 | 5.272 ± 1.743 | 2526 ± 262 | 2806 ± 174 | 3039 ± 195 |
| *Oh*-Away | 184.73 ± 37.36 | 31.33 ± 5.45 | 0.177 ± 0.025 | 7.019 ± 3.151 | 5.736 ± 1.109 | 6.315 ± 1.879 | 2143 ± 280 | 2359 ± 177 | 2468 ± 209 |
| *Oh*-Hybrid | 157.42 ± 32.90 | 30.67 ± 7.85 | 0.199 ± 0.034 | 5.444 ± 1.379 | 5.306 ± 1.298 | 4.694 ± 0.744 | 2060 ± 343 | 2372 ± 216 | 2498 ± 218 |
| *Ol*-Away | 127.48 ± 22.70 | 28.88 ± 5.28 | 0.236 ± 0.049 | 5.61 ± 2.62 | 4.45 ± 0.84 | 3.75 ± 0.83 | 2487 ± 286 | 2764 ± 242 | 2904 ± 294 |
| *Ol*-Stream | 121.37 ± 15.59 | 28.53 ± 5.99 | 0.241 ± 0.061 | 6.589 ± 3.290 | 4.156 ± 0.850 | 3.222 ± 0.741 | 2675 ± 257 | 2940 ± 213 | 3103 ± 204 |
